# Understanding Client Difficulties in Transdiagnostic Internet-Delivered Cognitive Behaviour Therapy: A Qualitative Analysis of Homework Reflections

**DOI:** 10.3390/jcm11144226

**Published:** 2022-07-21

**Authors:** Vanessa Peynenburg, Andrew Wilhelms, Ram Sapkota, Marcie Nugent, Katherine Owens, Nick Titov, Blake Dear, Heather Hadjisatvropoulos

**Affiliations:** 1 Department of Psychology, University of Regina, 3737 Wascana Parkway, Regina, SK S4S 0A2, Canada; vanessa.peynenburg@uregina.ca (V.P.); andrew.wilhelms@uregina.ca (A.W.); ram.sapkota@uregina.ca (R.S.); marcie.nugent@uregina.ca (M.N.); 2 Online Therapy Team, Regina Adult Mental Health Clinic, Saskatchewan Health Authority, 2110 Hamilton Street, Regina, SK S4P 2E3, Canada; katherine.owens@saskhealthauthority.ca; 3 MindSpot Clinic, MQ Health, Macquarie University, Sydney, NSW 2109, Australia; nick.titov@mq.edu.au; 4 eCentreClinic, School of Psychological Sciences, Macquarie University, Sydney, NSW 2109, Australia; blake.dear@mq.edu.au

**Keywords:** internet-delivered therapy, cognitive behaviour therapy, online therapy, digital mental health, homework, therapist support, e-health

## Abstract

Internet-delivered cognitive behaviour therapy (ICBT) is helpful for many clients, but less is known about the challenges clients face during ICBT, such as difficulties with skill practice, development, or maintenance. Understanding client difficulties can help therapists support clients with skill development and prevent treatment drop-out, but has not been systematically studied. This study included a conventional content analysis of clients’ responses to a homework reflection question about difficulties with lessons and skills. Data was drawn from a previously published trial of 301 clients who were randomly assigned to receive homework reflection questions during ICBT. A decreasing number of clients responded to the question about skill difficulties with each lesson. Clients who answered the question about difficulties were more engaged with ICBT (i.e., more lessons completed, logins, days enrolled in ICBT, and messages sent to therapists). Clients shared skill-specific challenges (including initial challenges and more advanced challenges), generic challenges (content or skills being cognitively draining or emotionally draining, contextual challenges, forgetfulness, limited time, and lack of familiarity with the skill), or no challenges. Thought challenging (59.6%) and graded exposure (57.5%) were associated with the greatest number of skill-specific challenges. Findings can help therapists anticipate and address common client challenges during ICBT.

## 1. Introduction

Cognitive behaviour therapy (CBT) is an effective psychotherapy that focuses on the development of cognitive and behavioural skills and is commonly used to treat anxiety disorders and depression [1]. Prospective clients face a variety of challenges related to stigma, location, and time that can act as a barrier to receiving face-to-face CBT [2]. Internet-delivered cognitive behaviour therapy (ICBT) is an alternative treatment option that can increase clients’ ability to access services, but some clients experience challenges with skill development during treatment. ICBT includes similar skills and content as clients would receive in face-to-face cognitive behaviour therapy (CBT), with all content delivered online typically in the form of lessons often combined with therapist support [3]. Meta-analyses of ICBT in routine care have identified Hedge’s g effects of 1.18 for reduced depression (95% CI 1.06–1.29) and 0.94 (95% CI 0.83–1.06) for reduced anxiety [2]. In general, deterioration side effects in ICBT appear to be low, with one review suggesting less than 3% of clients report deterioration [2]. Despite these promising findings, less is known about clients’ difficulties with skill development or behavioural change during ICBT. It may be beneficial to systematically monitor clients’ experiences throughout treatment so that therapists can attempt to facilitate skill acquisition with the aims of optimizing symptom improvement and sustaining engagement.

In traditional face-to-face cognitive behaviour therapy (CBT), therapists often assign and review homework exercises [4]. Homework reviews serve several purposes, including the following: reinforcing the importance of skills practiced outside of sessions, clarifying the objectives of assigned homework, problem-solving barriers to skill implementation, and offering feedback or positive reinforcement for clients’ efforts [5]. Additionally, homework review increases the likelihood of clients completing future homework exercises [6]. Clients in ICBT also typically receive homework assignments [3], although the nature of the review by the therapist varies widely among ICBT programs [7].

In therapist-assisted ICBT programs, one way that clients and therapists review homework assignments is by email. Soucy et al. [8] coded emails that clients sent to their therapists during ICBT and found that most clients asked fewer than two questions throughout the eight-week treatment. The most common questions were related to enhancing clients’ understanding of the materials and applying the skills from the lessons (46.72%). Of note, these questions were spontaneously reported by clients in their emails. Another approach to reviewing homework is to systematically ask clients to answer questions about their completion of homework in the form of homework reflection questionnaires (HWRQs) before clients proceed to the next ICBT lesson. Information gathered from these HWRQs can help therapists understand what challenges their clients are encountering during each lesson. It may be particularly helpful in cases when clients do not spontaneously share difficulties they are having in their emails. The role of including HWRQs on client engagement and client outcomes (regarding depression and anxiety) was recently examined in a factorial trial of ICBT [7]. Clients were randomly assigned to complete HWRQs or not and to receive either once-weekly or twice-weekly therapist support. Contrary to what was hypothesised, clients who were asked to complete HWRQs logged into the website fewer times and spent fewer days in the program overall. A possible explanation for this finding is that some clients felt the additional questions were too demanding, which acted as a deterrent to logging into the program. No benefits were found for the inclusion of HWRQs on improvements in depression or anxiety, so it was concluded that the inclusion of HWRQs may not be beneficial for improving either client engagement or outcomes. 

While the inclusion of HWRQs did not alter outcomes, there is a strong tradition in CBT of clients reflecting on homework. Further, therapists involved in the trial [7] reported several benefits to including HWRQs, such as increased knowledge about their clients and improved efficiency when conducting weekly check-ins with clients. Clients provided ratings on how much of the content they reviewed, level of effort in practicing the skills, difficulty practicing the skill, understanding of the lesson, and the helpfulness of the skill. As the course progressed, clients reported being less engaged with the content and putting in less effort over time. The content on de-arousal strategies was rated as the easiest to understand, and clients had the most difficulty practicing thought challenging exercises. These ratings provide information about what skills clients find the most challenging, but to date, there has been no exploration of the specific challenges faced by clients as they worked on lessons in ICBT.

The current study therefore included an evaluation of client responses to questions regarding difficulties with practicing CBT skills as they completed the five lesson transdiagnostic ICBT course. The primary objective of this study was to identify difficulties that clients encountered when completing ICBT, to provide an enhanced understanding of client response to ICBT, and to ultimately inform therapists’ practices for addressing clients’ challenges to learning and implementing CBT strategies. In this study, we aimed to answer four exploratory questions: (1) What percentage of clients will share difficulties in response to the five core lessons of the ICBT course? (2) Do clients who share difficulties differ from those who do not on measures of demographics, symptom severity, or treatment engagement? (3) What general difficulties do clients identify across all lessons? and (4) What specific difficulties do clients identify related to each lesson and accompanying skill? Understanding the characteristics of clients who share difficulties with homework in ICBT and the nature of those difficulties can help therapists be aware of what challenges their clients are likely to encounter during ICBT and how they can best support them.

## 2. Materials and Methods

### 2.1. Participants and Recruitment

This qualitative study was approved by the institutional research ethics board at the University of Regina. Data were obtained from a previously published randomized factorial trial (ClinicalTrials.gov NCT03957330 [7]) conducted between 2 May 2019 and 5 November 2019 investigating the inclusion of HWRQs (yes/no) and different levels of therapist support (once-/twice-weekly email contact). In this trial, ICBT resulted in moderate symptom improvement for depression (Cohen’s d: 0.69–0.78) and large improvement for anxiety (Cohen’s d: 0.82–0.91), that were maintained at the 24-week follow-up regardless of treatment condition. The current study specifically focussed on 301 clients who were assigned to the HWRQ condition and subsequently completed consent and started treatment. In this study, we were interested in how clients responded to an optional open-ended question about difficulties they experienced practicing the skill/skills from the previous lesson that was administered at five time points during the ICBT course (i.e., at the beginning of lessons 2–5 and at the beginning of the 8-week post-treatment questionnaires).

Of the 301 clients, 154 (51.2%) completed the question about difficulties they experienced in lesson 1 (CBT model), 151 (50.2%) completed the question for lesson 2 (thought challenging), 114 (37.9%) for lesson 3 (controlled breathing and activity planning), 87 (28.9%) for lesson 4 (graded exposure), and 57 (18.9%) for lesson 5 (goal setting and relapse prevention).

### 2.2. Intervention

All eligible clients were given access to the 8-week five lesson transdiagnostic ICBT program (the Wellbeing Course), developed by the eCentreClinic at Macquarie University [9] and licensed by the Online Therapy Unit. The course consists of five core lessons, each focusing on a different skill or strategy within CBT. Each lesson consists of 50 to 60 presentational slides and a downloadable guide that includes recommended homework assignments related to skills that correspond with concepts from the lesson slideshows. Case stories based on previous clients’ experiences are included in the lessons. The five lessons are made available to clients on a fixed schedule (lesson 1 opens immediately, lesson 2 opens at start of week 2, lesson 3 at start of week 4, lesson 4 at start of week 5, lesson 5 at start of week 7), pending completion of all previous lessons and any active survey. Automated emails are sent to notify clients when a lesson becomes available.

### 2.3. Measures

The HWRQs were administered at the beginning of lessons 2, 3, 4, and 5 and at post-treatment. Primary outcome measures included the Patient Health Questionnaire 9-item (PHQ-9 [10]) and Generalized Anxiety Disorder–7 (GAD-7 [11]) and are only reported on at pre-treatment in this study. The treatment website tracked client engagement, including the number of logins, days between first and last login, lessons accessed, messages client sent, messages therapist sent, and phone calls with the client. For a full list of the primary and secondary measures included in the factorial trial, see [7].

#### Homework Reflection Questionnaire (HWRQ)

Starting in the second lesson, clients in the HWRQ condition were asked at the beginning of each lesson to reflect on their experiences with the previous lesson’s homework activities. Clients provided ratings on how much of the previous lesson they were able to review, how much effort they put into working on the main skill from the previous lesson, the level of difficulty they had with working on the skills, how understandable the skills were, how helpful they found the skills, and to what extent they had continued to use the skills from previous lessons (see [6] for a summary of the ratings). Clients were also given an optional open-ended question (i.e., “Please share any difficulties you had with [skill]”) where they could share difficulties they had with the cycle of symptoms (lesson 1), thought challenging (lesson 2), controlled breathing and activity planning (lesson 3), graded exposure (lesson 4), and goal setting relapse prevention (lesson 5). The open-ended question about clients’ difficulties with the skills was the focus of our analyses in this paper.

### 2.4. Therapist Support

Clients were assigned to a therapist from either an ICBT clinic or a community mental health clinic. In both settings, the primary caseload of therapists was ICBT and all therapists received training and supervision in ICBT. As described in Hadjistavropoulos et al. [7], clients were randomized to receive either once-weekly or twice-weekly therapist support. Therapists contacted clients on a predetermined day each week in the once-weekly condition and on two predetermined days in the twice-weekly condition. Emails were composed based on reviewing client progress on the completion of lessons, any completed questionnaires (i.e., symptom questionnaires, HWRQ) and emails from clients. Therapists were expected to take 15 min per email although they had the flexibility to increase the amount of time spent when clinically indicated. Therapists were instructed to call the client by telephone when clinically indicated (i.e., increase in symptom scores of five points or more, endorsed suicidal ideation, client had not logged on in a week, or client requested phone contact).

### 2.5. Analyses 

#### 2.5.1. Quantitative Analyses

Analyses related to symptom changes over time are reported in Hadjistavropoulos et al. [7]. A combination of Chi-square analyses and one-way ANOVAs were conducted using SPSS Version 25 to examine any pre-treatment differences in demographic and clinical characteristics between clients who completed the HWRQ question on difficulties for at least one lesson and those who did not (see Table 1). To partially account for multiple comparisons, a *p*-value of 0.01 was considered statistically significant. Completion of the HWRQ question on difficulties was also compared between those who received once-weekly versus twice-weekly support. As no differences were found on this variable (χ^2^ _(1, 301)_ = 1.85, p = 0.17), no further analysis of this variable was undertaken. 

#### 2.5.2. Qualitative Analyses

Prior to reviewing the HWRQs, all responses to open-ended questions on treatment satisfaction were also examined to determine whether clients volunteered their opinions about the HWRQs. Clients did not make any explicit references to the HWRQs in their responses.

Conventional content analysis has been recommended when existing literature on a topic is limited [12]. Analysis of the HWRQ was conducted using NVivo 12, a qualitative analysis software by QSR International. The first author began by reviewing approximately 25% of the responses to the HWRQ question about client difficulties for each lesson to generate initial themes and form a codebook. After approximately 25% of the responses had been reviewed, no new themes were emerging in client responses. Two research associates then independently coded all responses to the HWRQ questions using the preliminary codebook. The reviewers could propose new themes if they felt the existing themes did not capture the clients’ responses. Proposed themes were discussed in a group meeting with the primary author and reviewers, a revised codebook was formed, and responses were recoded using the new themes. Once all the responses were coded, a report was generated in Nvivo 12 that included the percentage of responses for each code, as well as a list of all the responses that were assigned to a given code. The first author reviewed the report and identified any instances of disagreement. A third reviewer (R.S.) resolved any discrepancies.

## 3. Results

### 3.1. Client Characteristics

Pre-treatment characteristics of clients who were randomized to one of the HWRQ conditions (*n* = 301) are included in Table 1 (see [6] for a description of the full sample). The mean age of clients was 36.06 years (*SD* = 12.82) and the majority of clients were female (*n* = 224/301, 74.4%), married or common-law (*n* = 198/301, 65.8%), had at least some university education (*n* = 151/301, 50.2%), were employed (*n* = 211/301, 70.1%), and White (*n* = 276/301, 91.7%). At intake, 56.5% (*n* = 170/301) of clients were taking psychotropic medications and only 11.6% (*n* = 35/301) of clients did not have any scores in the clinical range on measures of generalized anxiety or depression. No significant differences were found on any demographic or clinical characteristics between clients who completed HWRQs and those who did not (*p* range: 0.05–93). 

### 3.2. Treatment Engagement

Treatment engagement is summarized in Table 1. Overall, clients who completed the HWRQ questions on difficulties were more engaged during the ICBT course in terms of being more likely to access lesson 4 (88.4% vs. 44.7%; χ^2^ _(1, 301)_ = 62.06, *p* < 0.001) and lesson 5 (79.1% vs. 38.2%; χ^2^ _(1, 301)_ = 44.37, *p* < 0.001), logging in more times (*t*(299) = −5.88, *p* < 0.001), having more days in the program between their first and last access (*t*(299) = −5.49, *p* < 0.001), and sending a greater number of messages to their therapists (*t*(299) = −6.33, *p* < 0.001) compared to clients who did not answer the HWRQ questions on difficulties. 

### 3.3. Qualitative Domains 

The qualitative analysis revealed that clients’ responses to the HWRQ item about difficulties with skills were found to fall under two main domains: challenges that were specific to the skill described in that lesson (“specific challenges”) and challenges that were experienced by clients across all five lessons (“generic challenges”). A third domain was the absence of challenges with the specific skill from that lesson (“no challenges”). Table 2 summarizes the proportion of responses that fell under each domain for each of the lessons. In general, skill-specific challenges were the most common type of challenge for lesson 1 (cycle of symptoms), 2 (thought challenging) and 4 (graded exposure). In the case of lesson 3 (controlled breathing and activity planning), it was most common for clients to report having no challenges. For lesson 5 on goal setting and relapse prevention, generic challenges were most commonly reported. 

#### 3.3.1. Specific Challenges

In reviewing clients’ difficulties with each of the five lessons, clients’ comments were coded as initial challenges versus intermediate challenges with the skills taught in the course, which were the following: cycle of symptoms (lesson 1), thought challenging (lesson 2), controlled breathing and activity planning (lesson 3), graded exposure (lesson 4), and relapse prevention (lesson 5). Initial challenges were defined as difficulties with getting started on a particular skill or troubles with seeing how the skill was relevant to their experience. Intermediate challenges were defined as difficulties with mastering the skill or generalizing it to different situations or environments. Table 3 includes example quotes of specific challenges for each of the five lessons, as well as the proportion of responses for each type of challenge.

##### Cycle of Symptoms

When reflecting on the cycle of symptoms among thoughts, behaviours and physical sensations, clients’ initial challenges (79.7%, 63/79) included difficulties with identifying their symptoms, triggers, or knowing where their cycle of symptoms started (e.g., “It took me some time to figure out what exactly my cycle of symptoms were” ^#13289^). For intermediate difficulties (20.3%, 16/79), clients commented on challenges with making connections between different types of symptoms or with what steps to take to address their symptoms after they gained an understanding of how they were connected (e.g., “It was fairly easy to identify what my unhelpful thoughts, physical symptoms, and unhelpful behaviours are but a bit difficult to figure out which of one category led to another category” ^#12815^).

##### Thought Challenging

Initial challenges related to thought challenging (57.8%, 52/90) included difficulties with identifying specific thoughts or finding it hard to recognize that the thought was unhelpful (e.g., “The biggest challenge I have at this point is clearly identifying the negative thought” ^#12539^). Intermediate challenges (42.2%, 38/90) included difficulties coming up with alternative thoughts, believing the alternative thoughts, or taking action in response to the thought (e.g., “I had trouble coming up with figuring out how to do something helpful as doing any of these things didn’t lessen the negative thoughts I had.” ^#13087^).

##### Activity Planning

Clients who had initial challenges (46.7%, 7/15) with activity planning described difficulties with identifying activities that they enjoy or challenges related to creating an activity schedule (e.g., “I have been thinking a lot about activity planning but I haven’t brought myself to fill in the schedule. I think I have anxiety around writing plans down.” ^#13914^). Intermediate challenges (53.3%, 8/15) included barriers to engaging in the activities, creating schedules with too many activities, or difficulties with implementing the schedules they had created (e.g., “Trying to do too much and then feeling bad I could not do it all.” ^#14045^).

##### Controlled Breathing

Initial challenges for controlled breathing (33.3%, 4/12) included clients feeling uncomfortable about trying the breathing strategy or thinking that controlled breathing will not be helpful for them (e.g., “I’m unsure it will be very helpful for me.” ^#13312^). Among clients who experienced intermediate challenges (66.6%, 8/12) with controlled breathing, they described having difficulty applying controlled breathing to different situations, experiencing physiological symptoms as a result of controlled breathing (e.g., light-headedness), concerns about others noticing that they were using the strategy, or with the pace of the breathing.

##### Graded Exposure

Clients’ initial challenges (82.0%, 41/50) with graded exposure included difficulties with identifying a target goal or behaviour that they would like to include in their exposure hierarchy or difficulty with breaking down their target goal or behaviour into smaller steps (e.g., “Coming up with something to work on.” ^#12563^). For clients who started working on graded exposure, intermediate challenges (18.0%, 9/50) included emotional difficulties (e.g., fear, anxiety) with progressing to the next step of their exposure stepladder, moving too quickly from one step to the next, being uncertain about when they should progress from one step to the next, or avoidance of the more challenging activities in their stepladder (e.g., “The hardest part of graded exposure is to move up the ladder. I find excuses to put things off once I get to the medium, hard, very hard.” ^#14096^).

##### Relapse Prevention

Clients who reported initial challenges (88.2%, 15/17) with relapse prevention described difficulties with identifying what their triggers or stressors were (e.g., “I have a hard time pinpointing what makes my symptoms flare up so find it challenging to know all that I’ll do to keep from relapsing” ^#13545^) or reported having difficulties getting started on their relapse prevention plan. Only two responses were identified as intermediate challenges (11.8%), with clients describing difficulties with recognizing a lapse once it had happened and knowing how to implement their relapse prevention plan (e.g., “It is easy to plan and think about, but probably harder to enact and notice the signs, especially the ones that are simply thoughts” ^#12450^).

#### 3.3.2. Generic Challenges across Lessons

Clients also described generic challenges that were similar across the five lessons that were unrelated to the specific skills described in each lesson, which accounted for between 24.5% (*n* = 37/151 for thought challenging) and 53.5% (*n* = 61/114 for managing physical symptoms) of clients’ responses as to what they were experiencing related to each lesson. The following themes were generated based on clients’ comments: finding the content/skills cognitively draining, finding the content/skills emotionally draining (e.g., client experienced an increase in symptoms or negative emotions when trying the skill), contextual challenges (e.g., challenges related to work, their health, relationships, or school), forgetting to use the skills when needed, limited time, and the client not being sufficiently familiar with the skill in order to use it (e.g., feeling like they need to review the content or practice more to understand the skill). Table 4 includes example quotes of generic quotes across the five lessons, as well as the proportion of responses for each type of challenge.

When reflecting on the lesson on the cycle of symptoms, the most common generic challenge was that it was emotionally draining (61.0%, 25/41). Clients noted how difficult it was to spend time paying attention to their symptoms and to recognise the impact their symptoms were having on their lives. For the lesson on thought challenging, the most common generic challenge was that the client forgot to use the skill (24.3%, 9/37) when experiencing unhelpful thoughts. Limited time (41.9%, 13/31) was the most common challenge for activity planning and clients described how they felt like they did not have sufficient time to add pleasant activities to their schedules. For controlled breathing, the most common generic challenge was forgetting to use the skill (60.0%, 18/30), with most clients recognizing in hindsight that controlled breathing would have been beneficial, but that they did not use it when they experienced symptoms of over-arousal. Similar to activity planning, clients commented on how limited time was a barrier to practicing graded exposure (30.8%, 8/26). Finally, contextual challenges or challenges related to stressors (e.g., illness, being on holidays, school) were identified as the most common generic challenge related to relapse prevention.

#### 3.3.3. No Challenges

A subset of clients reported that they did not experience any difficulties with practicing the skills from the different lessons. This type of comment was more common for participants to make about controlled breathing (36.8%, 42/114), relapse prevention (31.6%, 18/57), the cycle of symptoms (24.0%, 37/154), and activity planning (21.1%, 24/114). It was less frequently made with reference to thought challenging (15.9%, 24/151), and graded exposure (13.8%, 12/57). Of note, clients who made a positive or neutral comment about the skills were also included within this category.

## 4. Discussion

In face-to-face CBT, homework exercises play an important role in facilitating skill acquisition with therapists encouraged to review homework with clients to reinforce the importance of these skills [4,5]. In ICBT, where therapist contact is often asynchronous, it can be more challenging to engage in homework review with clients. One solution is to encourage clients to complete HWRQs on a weekly basis to elicit feedback about any difficulties clients have with practicing CBT skills [7]. From both a clinical and training perspective, it is helpful to gain an understanding of the challenges clients experience as they learn about and implement different skills during ICBT. The main objectives of the current study were the following: to identify whether clients who reported difficulties in the HWRQ differed from those who did not on any demographic or clinical variables or measures of engagement, and to identify the frequency and nature of skill-specific, as well as generic, challenges that clients experienced.

### 4.1. Client Difficulties

Only 51.2% (154/301) of clients described difficulties they had with skills in the first HWRQ and the percentage of clients who completed this question decreased with each lesson (Lesson 2: 50.2%; Lesson 3: 37.9%; Lesson 4: 28.9%; Lesson 5: 18.9%). Clients who reported difficulties on the HWRQs were not significantly different from those who did not in terms of demographic characteristics or symptom severity at pre-treatment. However, clients who reported difficulties did appear to be more engaged with ICBT, based on lesson completion, number of logins, days spent in the course, and number of messages sent to therapists. A possible explanation for this finding is that clients who were open about their difficulties with skill development were able to receive tailored support from their therapists to help them overcome their initial, intermediate, or generic challenges. It appeared that completion of the question and sharing difficulties might be a good sign. In guided ICBT for depression, it has been found that clients who took responsibility for their treatment and were “doers” (i.e., provided specific examples of how they applied the content to their lives and addressed barriers during treatment) were more successful in ICBT [13]. A further conclusion might be that clients who withheld sharing difficulties and did not complete the question were less engaged with treatment overall and might need more direct prompting from their therapists to reveal any challenges they were experiencing.

Skill-specific challenges were most common for thought challenging (59.6%, 90/151), followed by graded exposure (57.5%, 50/87), and the cycle of symptoms (51.3%, 79/154). On the other hand, skill-specific challenges were less common for activity planning and controlled breathing exercises (13.2% and 10.5%, respectively), which might be due to clients’ familiarity with these skills prior to ICBT (e.g., “Activity planning was something I do but without remembering why it was important” ^#14032^). Challenges that clients shared were generally classified as being initial basic rudimentary challenges rather than more complex challenges. In general, it seems therapists need to be prepared to help clients most often with initial challenges related to the skills, rather than more complex challenges. Although a high percentage of respondents noted specific challenges with later lesson skills, such as graded exposure (87.5%, 50/87) and relapse prevention (29.8%, 17/57), the overall number of clients who responded to the question about difficulties with the skills decreased from lesson to lesson. This trend may be explained by lower engagement with the HWRQ over time or lower engagement with ICBT generally over time, since the decrease in number of responses to the question about difficulties seemed to parallel lesson completion.

In addition to skill-specific challenges, clients described generic challenges consistently across skill areas (i.e., cycle of symptoms: 26.6%, thought challenging: 24.4%, activity planning: 27.2%, graded exposure: 29.9%, and relapse prevention: 36.8%). Generic challenges included difficulties with limited time, finding the content/skill emotionally draining, contextual challenges or challenges related to stressors, forgetting to use the skill, finding the content/skill cognitively draining, and lack of familiarity with the skill. Therapists should be mindful that based on the findings of this study, approximately one-quarter of clients would experience at least one of these generic challenges in any given lesson. The most common generic reasons did appear to change over time (cycle of symptoms: emotionally draining; thought challenging and controlled breathing: forgetting to use skill; activity planning, graded exposure, and relapse prevention: limited time), which could be helpful for therapists to know. Of note, limited time was the most common difficulty across lessons and was included in 20.4% (*n* = 38) of responses that were categorized as generic challenges. In other online psychological interventions, clients have shared similar concerns about limited time and external factors competing for their time [14]. While the reasons for treatment non-adherence in ICBT are not well understood, it is possible that clients’ difficulties with specific skills contributed to their likelihood of dropping out, especially in cases where therapist contact was asynchronous. By regularly including statements to normalize clients’ emotional difficulties with the skills, as well as offering encouragement for skills practice, therapists may help clients manage many of these emotional and practical challenges. Further, the challenges articulated by clients provided suggestions for how the intervention lessons, the automated communications around the lessons, and the support provided alongside them could be improved. For example, in the first lesson, more content may be needed to help clients understand their own cycle of symptoms and triggers, as well as information to normalise that this task could be emotionally draining. For the lesson on thought challenging, more prompts could be provided to help clients identify their thoughts, as well as reminders of the importance of utilizing thought challenging when they noticed an unhelpful thought. Comments about limited time were common for activity planning, graded exposure, and relapse prevention, so it might be helpful to bolster the rationale for these skills in the lesson content so that clients can recognize the importance of integrating the skills in their schedules.

In general, it was interesting to note that the challenges that clients reported in ICBT were the same types of challenges that were described in face-to-face CBT [15], although description of challenges in face-to-face CBT were also not commonly systematically studied [16,17]. In a study of clients experiencing psychosis, common homework challenges included low motivation, poor memory, difficulties understanding worksheets, and perceived relevance and benefits of completing the homework [16]. In another study, clients with depression were interviewed about their experiences with CBT in general and completing homework was the main aspect of treatment that clients described negatively, due to both emotional and practical reasons [15]. Clients described a fear of failure when completing homework, worry about doing it incorrectly, and difficulties completing worksheets on the cycle of symptoms and thought monitoring/challenging. These difficulties aligned with the initial difficulties described by clients for the cycle of symptoms and thought challenging in the current study.

Within the current study, 61.0% (25/41) of clients who reported generic difficulties with the cycle of symptoms found that creating a personal formulation of their symptoms was emotionally draining. This finding appeared consistent with how some clients experience case formulation in face-to-face CBT [18], which typically involves the client and therapist working collaboratively to identify interrelationships between symptom types [19]. In one study, clients were interviewed about their experiences with case formulation after completing CBT and four themes emerged: formulation led to greater understanding of problems, formulation helped clients feel understood and accepted, formulation led to an emotional shift, and formulation helped clients move forward [18]. A subtheme emerged within the theme of an emotional shift, whereby some clients noted that the formulation process resulted in distress because of increased awareness of their problems. One recommendation that followed from our study was that therapists should most likely forewarn clients that they might experience a temporary increase in distress or symptoms after working through their personal formulation, as normalization of clients’ feelings plays an important role in the formulation [20].

A promising finding from our study was that ICBT clients’ difficulties were similar to those reported in face-to-face CBT [15,16,17,18]. Clients did not report any difficulties that seemed specific to ICBT, which may suggest that HW engagement and skill development could be managed well within an online setting. When training therapists in ICBT, it is useful for them to know that clients will encounter similar barriers and challenges when completing HW as they do in face-to-face CBT.

### 4.2. Limitations and Future Directions

While this study provided a summary of clients’ experiences learning about and implementing common CBT skills, it did not include information about clients’ experiences with completing the HWRQs. Given that there was not any evidence for HWRQs improving symptoms of depression and anxiety [7], it was possible that some clients either did not find them helpful or found them aversive to complete, which may help explain low completion of the HWRQs overall. Future studies of HWRQs should include questions about clients’ experiences with completing HWRQs. Another limitation of this study was the low completion rate of the HWRQs, with the percentage of clients completing the question about difficulties with the skills decreasing from lesson to lesson. We were unable to conclude whether the lower response rate to later questions was a result of fewer clients experiencing difficulties in later lessons or if clients had become disengaged with the HWRQ over time. It was also not possible to know whether clients who did not complete HWRQ had similar experiences to those who did in terms of specific and generic challenges. A potential solution is to make the HWRQs mandatory for clients to complete to proceed with the next ICBT lesson, which would help to ensure that, at least for clients who continue with treatment, information is collected from a larger sample of clients.

### 4.3. Strengths

This study expands on the findings of a published factorial trial examining the impact of HWRQs on symptoms of depression and anxiety [7]. Although it is well documented that HW is an integral component of CBT [5,21], little is known about clients’ experiences completing homework during ICBT. The current study provides insight into clients’ experiences with each core skill in the ICBT course, and, by systematically asking all clients about their challenges with HW exercises, we were able to include responses from clients who might not have otherwise discussed these concerns in their messages to therapists. While the findings from the current study do not provide a complete picture of client difficulties, this study provides more information on client difficulties than previously available in the literature. Findings about skill-specific and generic challenges can help inform clinical practice, especially when training therapists in ICBT.

## 5. Conclusions

This qualitative study contributes to the literature by improving our understanding of clients’ difficulties with skill acquisition during ICBT. In this study, clients described a combination of skill-specific challenges and generic challenges across skills. Specific challenges were most common when describing experiences with thought challenging and graded exposure and were often associated with the early stages of skills development (i.e., identifying unhelpful thoughts or creating an exposure hierarchy). Activity planning and controlled breathing were associated with the fewest challenges. Generic challenges were fairly consistent across skill areas, with limited time being the most commonly reported challenge across skills. The findings of this study suggest that those who completed the difficulty questions were more engaged with ICBT, so completion of this question may also be a useful measure of engagement. This study helps therapists understand the most common generic challenges that clients encounter, as well as when they occur during treatment. When therapists are aware of the common challenges that clients encounter, they are better prepared to tailor their messages to both normalise client difficulties and offer suggestions for how to overcome barriers clients experience while completing homework exercises.

## Figures and Tables

**Table 1:** Pre-treatment patient characteristics by group.

Variable	All HWRQ Clients (*n* = 301)		Responded to HW Difficulties Question (*n* = 225)		Did Not Respond to HW Difficulties Question (*n* = 76)		Significance
	*n*	%	*n*	%	*n*	%	
Age							
Mean (*SD*)	36.06 (12.82)	-	36.90 (13.34)	-	33.58 (10.81)	-	*t*(299) = −1.96, *p* = 0.05
Range	18–88	-	18–88	-	18–65	-	
Gender							
Male	71	23.6	50	22.2	21	27.6	χ^2^ _(1, 295)_ = 0.85, *p* = 0.36
Female	224	74.4	170	75.6	54	71.1	
Two spirit	3	1.0	3	1.3	-	-	
Non-binary	1	0.3	1	0.4	-	-	
Not listed	1	0.3	-	-	1	1.3	
Prefers not to disclose	1	0.3	1	0.4	-	-	
Marital status							
Single/never married	77	25.6	56	24.9	21	27.6	χ^2^ _(1, 301)_ = 0.31, *p* = 0.86
Married/common-law	198	65.8	150	66.7	48	63.2	
Separated/divorced/widowed	26	8.6	19	8.4	7	9.2	
Education							
Less than high school	3	1.0	2	0.9	1	1.3	χ^2^ _(1, 301)_ = 0.88, *p* = 0.83
High school diploma	65	21.6	46	20.4	19	25.0	
Post high school certificate/diploma	82	27.2	63	28.0	19	25.0	
University education	151	50.2	114	50.7	37	48.7	
Employment status							
Employed part-time/full-time	211	70.1	157	69.8	54	71.1	χ^2^ _(1, 301)_ = 7.40, *p* = 0.19
Unemployed	18	6.0	13	5.8	5	6.6	
Homemaker	27	9.0	21	9.3	6	7.9	
Student	19	6.3	17	7.6	2	2.6	
Disability	14	4.7	7	3.1	7	9.2	
Retired	12	4.0	10	4.4	2	2.6	
Ethnicity							
White	276	91.7	203	90.2	73	96.1	χ^2^ _(1, 301)_ = 4.03, *p* = 0.13
Indigenous	14	4.7	11	4.9	3	3.9	
Other	11	3.6	11	4.9	-	-	
Location							
Large city (over 200,000)	138	45.8	105	46.7	33	43.4	χ^2^ _(1, 301)_ = 0.41, *p* = 0.81
Small to medium city	64	21.3	46	20.4	18	23.7	
Small rural location (under 10,000)	99	32.9	74	32.8	25	32.9	
Mental health characteristics							
Taking psychotropic medications	170	56.5	120	53.3	50	65.8	χ^2^ _(1, 301)_ = 36, *p* = 0.05
Pre-treatment GAD-7 ≧ 10	189	62.8	143	63.6	46	60.5	χ^2^ _(1, 301)_ = 0.22, *p* = 0.64
Pre-treatment PHQ-9 ≧ 10	203	67.4	152	67.6	51	67.1	χ^2^ _(1, 301)_ = 0.01, *p* = 0.94
Non clinical PHQ-9 and GAD-7 scores	35	11.6	27	12.0	8	10.5	χ^2^ _(1, 301)_ = 0.12, *p* = 0.73
Pre-treatment credibility Mean (*SD*)	21.16 (4.41)	-	21.14 (4.41)	-	21.20 (4.44)	-	*t*(299) = 0.09, *p* = 0.93
Treatment Engagement							
Accessed Lesson 4	233	77.4	199	88.4	34	44.7	χ^2^ _(1, 301)_ = 62.06, *p* < 0.001
Accessed Lesson 5	207	68.8	178	79.1	29	38.2	χ^2^ _(1, 301)_ = 44.37, *p* < 0.001
# of log-ins Mean (*SD*)	20.5 (13.2)	-	23.0 (13.3)	-	13.2 (9.8)	-	*t*(299) = −5.88, *p* < 0.001
Days in program until last access, Mean (*SD*)	67.4 (38.6)	-	74.2 (36.4)	-	47.3 (38.4)	-	*t*(299) = −5.49, *p* < 0.001
# of messages sent by client, Mean (*SD*)	4.3 (3.8)	-	5.1 (3.8)	-	2.1 (2.7)	-	*t*(299) = −6.33, *p* < 0.001
# of phone conversations with client, Mean (*SD*)	1.0 (1.1)	-	1.0 (1.1)	-	1.2 (1.3)	-	*t*(299) = 1.80, *p* = 0.07

Note. HWRQ = homework reflection questionnaires; HW = homework; GAD-7 = Generalized Anxiety Disorder-7; PHQ-9 = Patient Health Questionnaire-9.

**Table 2:** Frequency of skill-specific challenges, generic challenges and no challenges per skill.

Skills	Total Respondents (*n*)	Skill-Specific Challenges *n* (%)	Generic Challenges Across Skills *n* (%)	No Challenges *n* (%)
Cycle of symptoms L1	154	79 (51.3)	41 (26.6)	37 (24.0)
Thought challenging L2	151	90 (59.6)	37 (24.5)	24 (15.9)
Managing physical symptoms L3	114	27 (23.7)	61 (53.5)	66 (57.9)
Activity planning ^1^	114	15 (13.2)	31 (27.2)	24 (21.2)
Controlled breathing ^1^	114	12 (10.5)	30 (26.3)	42 (36.8)
Graded exposure L4	87	50 (57.5)	26 (29.9)	12 (13.8)
Relapse prevention/goal setting L5	57	17 (29.8)	21 (36.8)	18 (31.6)

Note. Each response could be coded into more than one theme, resulting in a larger number of coded units than total responses. L1 = Lesson 1; L2 = Lesson 2; L3 = Lesson 3; L4 = Lesson 4; L5 = Lesson 5. ^1^ Clients were asked about activity planning and controlled breathing in the same reflection questionnaire.

**Table 3:** Skill-specific challenges.

Domain/Theme	Example Quote	Total Respondents *n* (%)
Skill: Cycle of Symptoms		
Specific Challenges		*n* = 79
Initial challenges	“I find it hard to find cycles within my anxiety since it’s been feeling constant lately. The only time I get some relief is my finishing everything I need to do, and sitting down and reading until I start falling asleep.” #13947	63 (79.7)
Intermediate challenges	“I’ve identified my cycle of symptoms. But it’s very hard to change it. Lol I’ve been thinking a lot of realizing my symptoms and finding a way to change my thought process.” #12872	16 (20.3)
Skill: Thought Challenging		
Specific Challenges		*n* = 90
Initial challenges	“Finding the confidence to challenge thoughts was difficult. Often the thoughts we are supposed to be challenging have almost become familiar and comforting to wallow in, so standing up to them feels uncomfortable.” #13808	52 (57.8)
Intermediate challenges	“I found it hard because I think my unrealistic thoughts are realistic! I just find it difficult to change my thoughts into better ones.” #14124	38 (42.2)
Managing Physical Symptoms		
Skill: Activity Planning		
Specific Challenges		*n* = 15
Initial challenges	“I just have difficulty setting up a schedule and following it. Again, I try to plan for it but want it to be perfect and then feel that it won’t be.” #14172	7 (46.7)
Intermediate challenges	“Because I worry a lot about timing and not having enough to get done what I want to, activity planning was a little bit of a struggle for me because I’d feel defeated if I wasn’t able to do what I set out to do” #13248	8 (53.3)
Skill: Controlled Breathing		*n* = 12
Initial challenges	“just calming down enough to breath slower” #13125	4 (33.3)
Intermediate challenges	“Controlled breathing is good for preparation when I’m going into a stressful situation but if I am caught in an unexpected situation it is difficult because I’m scared people will notice it and that will make me feel more nervous.” #12886	8 (66.6)
Skill: Graded Exposure		
Specific Challenges		*n* = 50
Initial challenges	“I found I had a hard time coming up with a stepladder approach to my goals at first, and it took a lot of time thinking and reflecting to be able to come up with something I felt was practical.” #13952	41 (82.0)
Intermediate challenges	“The hardest part of graded exposure was to move up the ladder. I find excuses to put things off once I get to the medium, hard, very hard.” #14096	9 (18.0)
Skill: Relapse Prevention		
Specific Challenges		*n* = 17
Initial challenges	“It was tough to plan for possible lapses. I had never really thought about being prepared beforehand for something like that and always took a reactionary approach instead of being proactive.” #14102	15 (88.2)
Intermediate challenges	“It is easy to plan and think about, but probably harder to enact and notice the signs, especially the ones that are simply thoughts.” #12450	2 (11.8)

**Table 4:** Generic challenges per skill and no challenges.

Domain/theme	Example Quote	Cycle of Symptoms *n* (%)	Thought Challenging *n* (%)	Managing Physical Symptoms *n* (%)		Graded Exposure *n* (%)	Relapse Prevention *n* (%)	Across Lessons *n* (%)
				Activity Planning	Controlled Breathing			
Generic Challenges		*n* = 41	*n* = 37	*n* = 31	*n* = 30	*n* = 26	*n* = 21	*n =* 186
Cognitively Draining	“I feel my concentration is so poor that even concentrating on making the connections between my cycles of symptoms presents a challenge.” #12886	5 (12.2)	3 (8.1)	6 (19.4)	4 (13.3)	4 (15.4)	4 (19.0)	26 (14.0)
Emotionally Draining	“It was difficult because it made me feel bad, like I was a bad person. It made me feel like, wow, I can see that I’m thinking unhelpful thoughts a lot but I don’t know how to stop because I believe that they are true.” #12431	25 (61.0)	6 (16.2)	1 (3.2)	0 (0)	3 (11.5)	0 (0)	35 (18.8)
Contextual challenges or challenges related to stressors	“I have been sick with a kidney infection the last two weeks and hospitalized.” #13063	3 (7.3)	7 (18.9)	8 (25.8)	1 (3.3)	7 (26.9)	8 (38.1)	34 (18.3)
Forgot to use skills	“Just remembering to do it when having a difficult time” #13545	1 (2.4)	9 (24.3)	3 (9.7)	18 (60.0)	2 (7.7)	0 (0)	33 (17.7)
Limited Time	“Busy schedule lately has made some things difficult to keep up with.” #13261	5 (12.2)	5 (13.5)	13 (41.9)	0 (0)	8 (30.8)	7 (33.3)	38 (20.4)
Not familiar with skill	“I just started the controlled breathing today so need to keep practicing it.” #12539	2 (4.9)	7 (18.9)	0 (0)	7 (23.3)	2 (7.7)	2 (9.5)	20 (10.8)
**No Challenges**	“I didnt really have any difficulty. It just surprised me that all of the symptoms that i have, all connect in some way.” #12773	*n* = 37	*n* = 24	*n* = 24	*n* = 42	*n* = 12	*n* = 18	*n* = 157

## Data Availability

Not applicable.
